# A Nanocomposite Sol-Gel Film Based on PbS Quantum Dots Embedded into an Amorphous Host Inorganic Matrix

**DOI:** 10.3390/ma16227105

**Published:** 2023-11-09

**Authors:** Mihail Elisa, Bogdan Alexandru Sava, Mihai Eftimie, Adrian Ionut Nicoara, Ileana Cristina Vasiliu, Madalin Ion Rusu, Cristina Bartha, Monica Enculescu, Andrei Cristian Kuncser, Mihai Oane, César Elosúa Aguado, Diego López-Torres

**Affiliations:** 1 National Institute of R&D for Optoelectronics-INOE 2000, 409 Atomistilor Str., 077125 Magurele, Romania; astatin18@yahoo.com (M.E.); icvasiliu@inoe.ro (I.C.V.); madalin@inoe.ro (M.I.R.); 2 National Institute for Laser, Plasma and Radiation Physics, 409 Atomistilor Str., 077125 Magurele, Romania; mihai.oane@inflpr.ro; 3 Faculty of Chemical Engineering and Biotechnologies, University Politehnica of Bucharest, 1 Gheorghe Polizu Str., 011061 Bucharest, Romania; 4 National Institute of Materials Physics, Atomistilor 405 A, 077125 Magurele, Romania; cristinavals@yahoo.com (C.B.); mdatcu@infim.ro (M.E.); andrei.kuncser@infim.ro (A.C.K.); 5 Department of Electrical, Electronic and Communications Engineering, Public University of Navarra, E-31006 Pamplona, Spain; cesar.elosua@unavarra.es (C.E.A.); diego.lopez@unavarra.es (D.L.-T.); 6 Institute of Smart Cities (ISC), Public University of Navarra, E-31006 Pamplona, Spain

**Keywords:** PbS, quantum dots, sol-gel method, amorphous material

## Abstract

In this study, a sol-gel film based on lead sulfide (PbS) quantum dots incorporated into a host network was synthesized as a special nanostructured composite material with potential applications in temperature sensor systems. This work dealt with the optical, structural, and morphological properties of a representative PbS quantum dot (QD)-containing thin film belonging to the Al_2_O_3_–SiO_2_–P_2_O_5_ system. The film was prepared using the sol-gel method combined with the spin coating technique, starting from a precursor solution containing a suspension of PbS QDs in toluene with a narrow size distribution and coated on a glass substrate in a multilayer process, followed by annealing of each deposited layer. The size (approximately 10 nm) of the lead sulfide nanocrystallites was validated by XRD and by the quantum confinement effect based on the band gap value and by TEM results. The photoluminescence peak of 1505 nm was very close to that of the precursor PbS QD solution, which demonstrated that the synthesis route of the film preserved the optical emission characteristic of the PbS QDs. The photoluminescence of the lead sulfide QD-containing film in the near infrared domain demonstrates that this material is a promising candidate for future sensing applications in temperature monitoring.

## 1. Introduction

Lead chalcogenide colloidal quantum dots (CQDs) are the most commonly used type of CQDs for photovoltaic and light-emitting devices due to their adjustable band gap (from visible to near infrared), high dielectric constant, intense light absorption, and good stability [1,2]. PbS quantum dots are especially interesting for optoelectronic applications and spectroscopic techniques, including photoluminescence, photodiodes, and solar cells [3]. A recent study related to the doping of PbS QD films using organic molecules to improve carrier mobility was applied in PbS field-effect transistors using SiO_2_ as the dielectric gate. Doping reduced the contact resistance of the device and improved the device’s capabilities [4]. PbS QD-doped TiO_2_ nanotubes, as a thin layer, were used in planar perovskite solar cells to improve efficiency by acting as an electron transport layer (ETL) as compared with TiO_2_ ETL [5]. It was demonstrated that the mobility of charges in the case of PbS QD films used in solar cells, photodetectors, and phototransistors could be improved by mixing with small amounts of PbSe QDs, due to the higher exciton radius of the latter [6]. The effect of perfluorocarbon compound (trifluoromethyl) coating on the electrical properties of PbS QDs in thin film transistors was reported in [7]. Hybrid solar cells of the ITO/Al:ZnO/PbS QDs/P3HT&PCBM/Ag type were reported and the effect of PbS QD size on the photovoltaic properties was demonstrated [8]. Both Al-doped ZnO and the intermediate PbS QD film increased the power conversion efficiency to 2.45%. It has been shown that optimization of the size and doping concentration of the ZnO window layer could improve the power conversion efficiency of PbS QD film-based solar cells [9]. A ZnO window film was deposited by using the layer-by-layer (LBL) sol-gel method [9] and the effects of reducing surface defects and improving the heterojunction quality of the solar cells were demonstrated.

A recent study related to intraband transitions in n-doped PbS CQD films, spin coated layer-by-layer on silicon substrates and dependent on temperature and dopant nanoparticle size, has shown that the substrates have applications in mid- and long-wavelength infrared photodetectors and light-emitting devices [10]. The dependence of the optical absorption and emission of PbS CQD films that were created using the layer-by-layer (LbL) technique, on size, temperature, and QD coupling, was reported in [11]. The authors showed that luminescence quenching increased with inter-dot coupling and, also, a negative dependence of energy band gap with temperature. PbS CQDs have been used as a novel image sensor for the IR domain, based on a combination of a quantum dot photodiode and a Si-based CMOS circuit [12]. The dependences of the emission features (intensity, wavelength position, and full width at half maximum) of PbS QDs on temperature in the range of 10–300 K and excitation strength have been investigated [13]. An investigation of carrier localization, recombination, and carrier–phonon interactions has been performed in the case of PbS QDs and PbS/MnTe QDs to explain the dependence of luminescence on temperature in the range of 10–300 K [14].

PbS QD-doped glass prepared using the melting method at 1400 °C was recently reported, and potential applications in optical thermometers were discussed based on the temperature-dependent photoluminescence (PL) spectra [15,16].

Our research focuses on the development of a low-cost, low-temperature, green technology based on the sol-gel method. We report on a study of the optical, structural, and morphological properties of a sol-gel nanostructured thin film consisting of a complex amorphous phase belonging to the Al_2_O_3_–SiO_2_–P_2_O_5_ oxide system embedding a crystalline phase based on PbS QDs. We aimed to exploit this inorganic matrix due to its chemical and thermal stability and its ability to prevent embedded PbS oxidation in normal atmosphere. Correlations between different properties were investigated, and the size of the lead sulfide nanocrystalline particles was determined by various methods and correlated to the lead sulfide QD dimension from the acquired precursor reagent. Precise control of the size and spatial distribution of PbS QDs is the key for applications; the Al_2_O_3_–SiO_2_–P_2_O_5_ film can provide a homogeneous porous structure with a narrow size range and the capacity to embed well-distributed small nanocrystals into the matrix. The proposed preparation method of the nanostructured film was verified to preserve the initial photoluminescence response of the PbS QDs from the suspension to the glassy film.

The composite material can be considered to be an attractive candidate for future applications in temperature-sensing devices.

## 2. Materials and Methods

### 2.1. Synthesis of the Film

The PbS QD-containing film belonging to the Al_2_O_3_–SiO_2_–P_2_O_5_ system was synthesized by using the sol-gel method combined with the spin coating technique [17]. The precursors used were aluminum acetylacetonate (C_15_H_21_O_6_Al), precursor of Al_2_O_3_; tetraethoxysilane (Si(OC_2_H_5_)_4_), precursor of SiO_2_; and triethylphosphate-(C_6_H_15_O_4_P), precursor of P_2_O_5_. Other chemical reagents used were ethanol (C_2_H_5_OH) as a reaction medium and monoethanolamine (C_2_H_7_NO) to stimulate the gelation chemical reactions. Lead sulfide QDs with a coating of oleic acid and dissipated in toluene were also used, with a concentration of 10 mg/mL and emission at approximately 1500 nm (Sigma-Aldrich PbS core-type quantum dots, code 900728). The analytical grade precursors were acquired from Sigma-Aldrich, St. Louis, MO, USA. The starting reagent molar ratios are presented in [17]. The solution, with pH of approximately 8, was synthesized using 5.9895 mL of starting reagents composed of tetraethoxysilane, triethyl phosphate, ethanol, monoethanolamine, and 5 mL of lead sulfide QD solution. The final solution was maintained at an ambient temperature under continuous magnetic spinning, for 2 h, to attain improved incorporation of the precursors, and then coating of the spinning glass substrates of approximately 25 mm size was performed [18,19,20,21]. The film synthesis occurred at 2000 rpm, for 20 s, 50 layers, and each layer was heat treated on an electrical plate at 150 °C, for 2 min, to release ethanol, water, and organic compounds from the film and to catalyze the amorphous network constitution. The vacuum atmosphere prevented lead sulfide QD oxidation, which could modify the optical properties of the film. The amorphous host network was in a steady state from a chemical and thermal point of view, with a chemical composition that could attain reliable and reproducible properties [17]. A film without PbS QDs was also deposited on a glass substrate, maintaining the same molar ratios of the precursors and similar synthesis parameters as those for the PbS QD-containing film.

### 2.2. Measurements

Film deposition was performed using a spin coater (WS-650SZ, Laurel Spinner, Laurell Technologies Corporation, North Wales, PA, USA) on a glass substrate (2.5 × 2.5 mm^2^) that had been previously chemically cleansed. For the XRD measurements, a BRUKER D8 ADVANCE (Billerica, MA, USA) X-ray diffractometer (CuKα, *λ* = 1.5405 Å) was used. The X-ray pattern was acquired at room temperature, with a step of 0.020° and 5 s integration time, and the scanning was performed between 5° and 70° (2θ range). The ICDD Powder Diffraction database was used for phase identification [22].

A Lambda 1050 spectrophotometer, (PerkinElmer, Waltham, MA, USA), in the 320–2500 nm range, was used for the optical absorption measurements, with a measurement error of ±0.03%.

The photoluminescence spectra, in the 850–1550 nm range, were recorded using a spectrofluorometer FluoroLog-3, HORIBA Jobin Yvone S.A.S. (Paris, France). The excitation wavelength was 850 nm, from a 450 W Xe lamp source, and the measurement error was ±0.5 nm.

The Raman spectra, in the 100–2000 cm^−1^ range, were collected with the aid of a LabRam HR Evolution HORIBA instrument, (Palaiseau, France), acquisition time 2 s, accumulation 20, 514 nm laser, with a hole diameter of 100 μm, objective 50×, grating of 600 gr/mm, and a range between 100 and 16,000 cm^−1^, and the measurement error was ±0.5 cm^−1^.

The morphology and elemental composition of the prepared samples was analyzed using a Carl Zeiss Gemini 500 field emission scanning electron microscope (FESEM) (Carl Zeiss, Oberkochen, Germany) for morphological measurements and a Bruker (Bruker, Bremen, Germany) Quantax energy dispersive X-ray spectrometer (EDS) with an energy resolution of 129 eV and Peltier cooling. The FESEM surface evaluation was conducted without metallic covering of the deposited film. Freshly cleaved samples were used for the evaluation of the film thickness in cross section.

The transmission electron microscopy (TEM) investigations were obtained using a JEM ARM200F instrument (JEOL, Tokyo, Japan), equipped with an ultra-high-resolution pole piece. The TEM specimens were prepared by the powder method, i.e., scratching the film from the substrate, dispersing the resulting material in ethanol, and drop-casting the as-obtained suspension on a standard TEM grid (lacey C, 200 mesh copper grid).

An atomic force microscope (AFM), model XE100 from the Park System Company (Suwon, Korea) was used to acquire the AFM images. Two distinct areas of 40 µm × 40 µm and 5 µm × 5 µm were scanned. The measurement error was approximately ±5%.

## 3. Results and Discussion

### 3.1. X-ray Diffraction Analysis (XRD)

**Figure 1:**
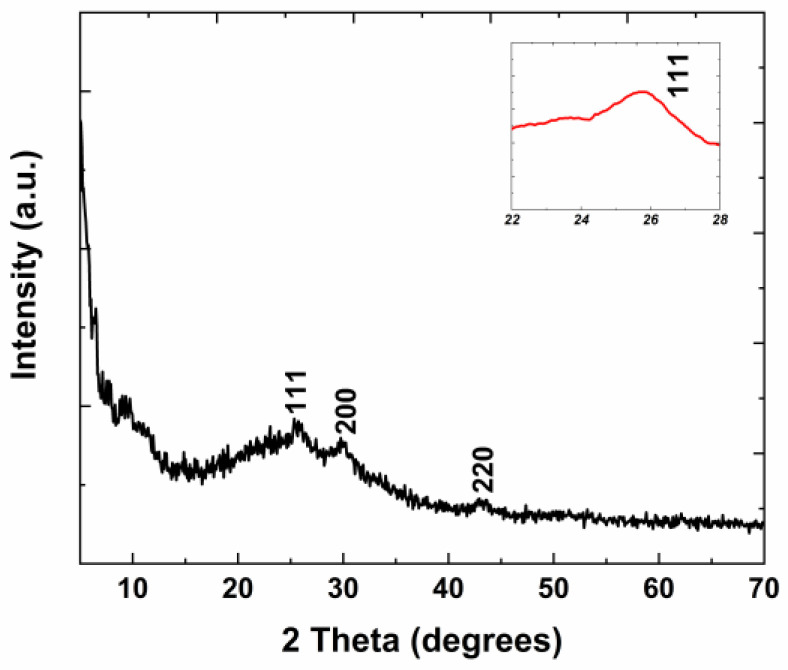
XRD pattern of the lead sulfide QD-containing film. The inset presents the (111) diffraction peak.

The XRD pattern of the PbS QD-containing film is depicted in Figure 1. For the PbS QD-containing film, the amorphous character of the material can be observed, corresponding to the inorganic host matrix from the Al_2_O_3_–SiO_2_–P_2_O_5_ system. At the same time, a cubic phase of PbS (space group Fm-3m) can be identified according to the PDF card 04-004-5729. Diffraction peaks at 2θ = 25.8°, 29.77°, and 42.90° can be observed, which correspond to the <111>, <200>, and <220> planes of the cubic phase of PbS, respectively.

The dopant nanoparticle dimension was established using the Scherrer formula, applied to the (111) reflection at 25.61° (2θ) [17,18,23], as Equation (1):

(1) $$d=\frac{k\lambda }{\Delta cos\theta }$$

where *k* is the Scherrer constant (the shape factor has a typical value of about 0.9), *λ* is the wavelength of the incident X-ray beam (λ_CuKα1_ = 1.5406 Å), 2θ is the peak position of the reflection, and Δ is the full width at half maximum of the reflection.

The dimension of the lead sulfide nanoparticles was estimated to be approximately 14.7 nm, comparable to the size of 7.2 nm, corresponding to lead sulfide QDs from the precursor solution in toluene (Sigma-Aldrich technical specification).

### 3.2. Optical Properties

#### 3.2.1. Optical Absorption

**Figure 2:**
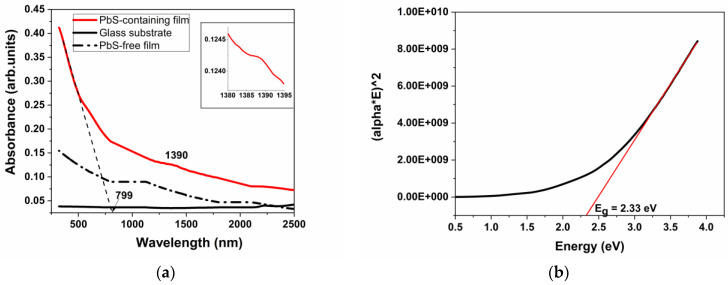
(**a**) Optical absorbance of the PbS QD-containing film, PbS QD-free film, and glass substrate (the inset shows the absorption band at 1390 nm); (**b**) optical band gap value of PbS QD-containing film, determined by Tauc’s law (graphical determination).

Figure 2a shows the optical absorbance of the following materials: glass substrate, PbS QD-free film, and lead sulfide QD-containing film. There are some optical losses due to scattering by quantum dots. As noted by other authors, in the case of quantum dots of other sulfides or selenides with dimensions similar to our PbS QDs of several nm, the value of such losses is two orders of magnitude lower than the absorption [24]. Other authors [25] have shown that the number of molecular layers of QDs does not change the performance of LEDs, which proves that the scattering effect is too weak to affect the performance of white LEDs, since MLs mainly affect the scattering of QDs [26]. Since the scattering losses were much smaller than the absorption, they were neglected.

Reduced absorbance of the glass substrate is observed, which increases in the case of the PbS QD-free film, and related to the lead sulfide QD-containing film a significant increase in optical absorption is observed, starting from the UV to the VIS and NIR regions. An absorption peak is found at 1390 nm, only for the sample with PbS QD-containing film, as shown in the inset of Figure 2a, which is assigned to the first exciton transition. The same absorption peak was reported by Ramiro et al. [10] for PbS QDs with a size of approximately 5.2 nm. The cutoff wavelength inferred from Figure 2a is λ_cutt-off_ = 799 nm. In the case of the PbS QD-free film, a decrease in optical absorbance from the UV to the VIS and NIR regions is observed, which is lower than the absorbance of the PbS QD-containing film.

The band gap energy, *E_g_*, of the lead sulfide QDs from the deposited film can be calculated as the total of the exciton binding and exciton peak energy, inferred by the absorbance spectrum. It is approximated that the lead sulfide QD exciton binding energy is four times higher than the lead sulfide exciton binding energy as bulk material, i.e., *E^bulk^* [27]. For lead sulfide QDs, the exciton binding energy *E^bulk^* (lead sulfide) is 3.968 meV [28]. Consequently, in the case of an electron effective mass of 0.085 *m*_0_ (*m*_0_ is the free electron mass), equal to the hole effective mass [29,30], the lead sulfide QD exciton binding energy is 15.872 meV. The energy of the exciton peak inferred by the absorbance spectrum is 0.892 eV; and therefore, the band gap energy *E_g_*, is 0.907 eV.

In compliance with [16,31], the dependence of the band gap energy on the dimension of the semiconductor QDs is calculated using Equation (2):

(2) $$E_{g}^{QDs}=E_{g}^{Bulk}+\frac{h^{2}}{2m^{*\;}d^{2}}$$

(3) $$m^{*}=\frac{m_{1}m_{2}}{m_{1}+m_{2}}$$

where *E_g_^QDs^* is the effective band gap energy of lead sulfide QDs; *E_g_^Bulk^* is the band gap energy of bulk lead sulfide, i.e., 0.41 eV [1]; m* is the exciton reduced mass [31]; *m*_1_ is the electron effective mass; *m*_2_ is the hole effective mass; *m*_0_ is the free electron mass (Equation (3)); *h* is Planck’s constant; and *d* is the size of the lead sulfide QDs [17]. The lead sulfide QD size, *d*, which is calculated from Equation (2), based on Equation (3), using *E_g_* = 0.907 eV, is approximately 8.45 nm, close to the value inferred by the XRD analysis, i.e., 14.7 nm, and to the value of 7.2 nm for the PbS nanocrystallites from the precursor solution. Therefore, the quantum confinement phenomenon, which is based on dependence of the band gap energy on the QD dimension, is valid in the case of lead sulfide QD-containing film, taking into consideration that the QD dimension is smaller than the lead sulfide exciton Bohr radius, i.e., 18 nm [17,27,28].

The graphical estimation of *E_g_* for the lead sulfide QD-containing film is presented in [18,32,33]. The Mott and Davis/Tauc Equation (4) was applied to graphically determine the optical band gap energy, i.e., *E_g_* (Figure 2b). Consequently, Equation (4) [16,33],

(4) $$\alpha h\nu ={\left(h\nu -E_{g}\right)}^{n},$$

allows the band gap energy determination, *E_g_*, where *α* is the absorption coefficient depending on wavelength, *h* is the Planck’s constant, *ν* is the light frequency, and *n* is ½ for the allowed direct electron transition and 2 for the allowed indirect electron transition, from the valence to the conduction band [34]. The absorption coefficient, *α*, is calculated using Equation (5) [35] as follows:

(5) $$\;\;\;\;\;\;\alpha =\frac{A\times 2.303}{x}.$$

where *A* is the optical absorbance depending on wavelength, as shown in Figure 2a, and *x* is the film thickness, i.e., 0.325 µm, as observed from the SEM analysis, in cross section. The absorption coefficient, *α*, is deduced from Equation (5) and is between 5134 and 29143 cm^−1^.

For the lead sulfide PbS QD-containing film, the value of *n* is ½, for the nanocrystalline semiconductor nominal amount. The energy band gap value, *E_g_*, is estimated by extrapolating the linear side of the graph to the zero absorbance, where *αhν* = 0 and, consequently, *E_g_* = 2.33 eV (Figure 2b). The wavelength conforming to *E_g_* = 2.33 eV is *λ_g_* = 532 nm. For *E_g_* = 2.33 eV and using Equation (2), the lead sulfide dimension is calculated, i.e., *d* = 2.78 nm, which is comparatively near the value inferred by the XRD analysis, i.e., 14.7 nm, and the value of 8.45 nm corresponding to *E_g_* = 0.907 eV related to the exciton peak energy.

The discrepancy related to the band gap energy value calculated from the energy of the first exciton, i.e., 0.907 eV, and the value calculated based on the Mott and Davis/Tauc law, i.e., 2.33 eV, can be clarified taking into consideration the type of the lead sulfide QDs-containing film. Therefore, the deposited film is a complex material containing an amorphous host network (Al_2_O_3_–SiO_2_–P_2_O_5_) and PbS QD crystalline nanoparticles, as shown in the XRD pattern (Figure 1). The graphical calculation of *E_g_*, applying the Mott and Davis/Tauc law, only considers the nanocrystalline phase composed of prevalent PbS quantum dots, taking *n* = 1/2. It does not consider the amorphous phase of the host network consisting of Al_2_O_3_–SiO_2_–P_2_O_5_ compounds in a lower nominal amount, corresponding to *n* = 2. Actually, in the case of this complex material, the *n* value cannot be precisely estimated, as it depends on the type and amount of the component phases.

**Figure 3:**
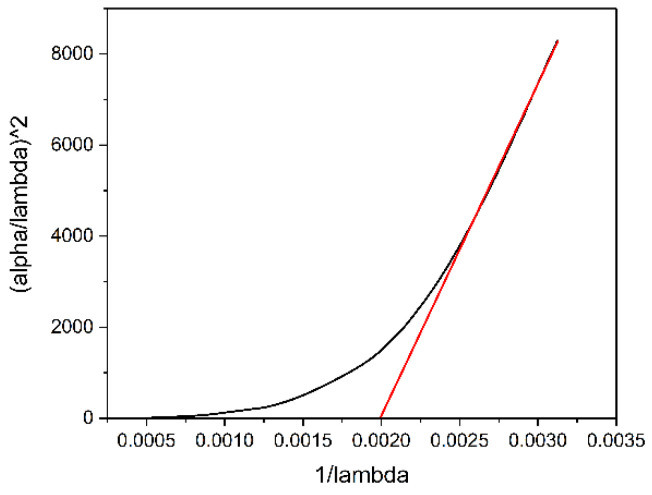
The dependence of (*α/λ*)^2^ on 1*/λ* for ASF validation, in the case of PbS QD-containing film.

In order to check the credibility of the graphically calculated *E_g_* and *λ_g_* values, the absorption spectrum fitting (ASF) method was used [18,21,33]. Thereby, $E_{opt}^{ASF}$ was calculated and compared to the *E_g_* value deduced from the Mott and Davis/Tauc law. Consequently, Tauc’s law is expressed as Equation (6) [18,33],

(6) $$\frac{\alpha hc}{\lambda }={\left(\frac{hc}{\lambda }-E_{g}\right)}^{1/2},$$

where *α* is the absorption coefficient; *h* is the Planck’s constant; *c* is the light speed in vacuum, equal to 3 × 10^8^ m/s; *λ* is the wavelength; and *E_g_* is the band gap energy. Hence, the function ${\left(\alpha /\lambda \right)}^{2}=f\left(\frac{1}{\lambda }\right)$ is graphically shown in Figure 3.

In the case of the resulted graph, the tangent to the linear side intersects the abscissa axis at a point, 1/*λ_g_*_(*ASF*)_. Thus, it is possible to deduce the *λ_g_*_(*ASF*)_ value. This value is comparatively analyzed with that calculated from Tauc’s law. Thus, 1*/λ_g_*_(*ASF*)_ = 0.002 and *λ_g_*_(*ASF*)_ = 500 nm, which is similar to that calculated from Tauc’s law, *λ_g_* = 532 nm. Further on, Equation (7) [18,33],

(7) $$E_{opt}^{ASF}=\frac{1240}{\lambda_{g}},$$

is used to calculate $E_{opt}^{ASF}$, which, in this case, is 2.48 eV. This is comparatively close to 2.33 eV, which is graphically calculated using Tauc’s law.

#### 3.2.2. Optical Emission

**Figure 4:**
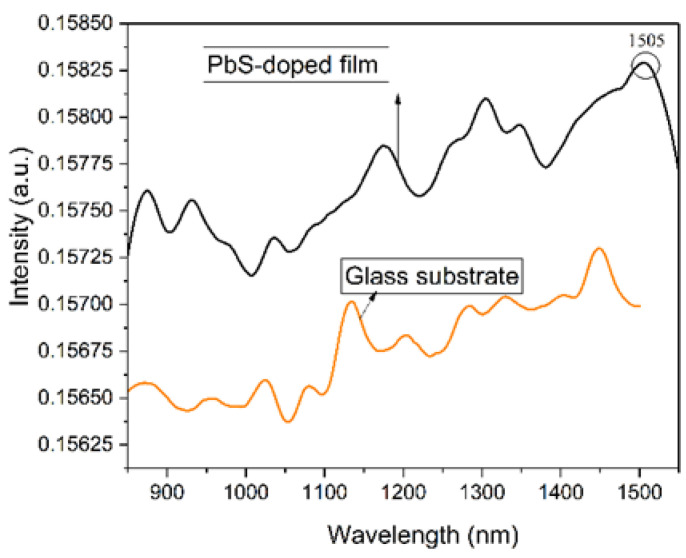
Photoluminescence of the lead sulfide QDs-containing film and glass substrate, recorded at 800 nm excitation.

The photoluminescence of the glass substrate and lead sulfide QD-containing film, in the range 850–1550 nm, is shown in Figure 4, collected at 800 nm beam excitation. No emission peak is found around 1500 nm for the glass substrate as compared with the lead sulfide QD-containing film where a photoluminescence is noticed at 1505 nm. The position of the emission maximum for the lead sulfide QD-containing film is similar to the photoluminescence maximum position of the lead sulfide QD precursor solution, certifying the protective role of the host network.

The electron/hole trap states of lead sulfide nanoparticles and the defect states at the boundary between the lead sulfide nanoparticles and the host network exhibit a significant influence on the emission characteristics of semiconductor dopant QDs. This influence states that the surface trap and defect states are intensively dependent on the dimension of the QDs [36]. Once the lead sulfide nanoparticles are excited by the 800 nm beam, a transition takes place between ^1^*S_h_* (hole ground state from the valence band, distinguished by energy, *E_h_*_0_) and ^1^*S_e_* (electron ground state from the conduction band, distinguished by energy *E_v_*_0_), conforming to the first exciton maximum, from 1390 nm in the absorbance spectrum.

Owing to the quantum confinement phenomenon, for quantum dots having a dimension smaller than the Bohr exciton radius (18 nm for lead sulfide), the energy levels of the hole states from the valence band and the electron states from the conduction band are quantized.

The photogenerated electron will be trapped by the electron trap states (ETSs) situated under the ^1^*S_e_* level, at high energy levels of the defect states (DSs). Further on, a radiative transition from the ETS levels takes place to the ^1^*S_h_* level, conforming to the emission maximum from 1505 nm. Thus, a wavelength shift (Stokes line) appears, related to the exciton peak placed at 1390 nm. Once the quantum dot dimension is increasing, conforming to Equation (2), the band gap energy value is diminishing, and the ETSs are situated very close or are superimposed by level ^1^*S_e_*, both situated at low energy levels from the DSs. Thus, the radiative transition from the ETSs to the ^1^*S_h_* level will take place and the respective energy will be reduced, the Stokes shift will decrease until no Stokes shift is found. Defect states are found in the host network such as non-bridging oxygen atoms, structural modifiers, and other disordered structures situated at the boundary with lead sulfide quantum dots [36,37].

### 3.3. Raman Spectroscopy

**Figure 5:**
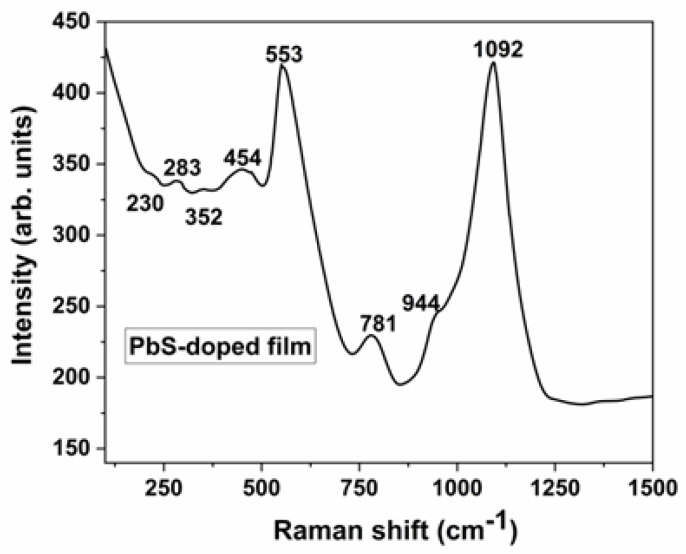
Raman spectrum of the lead sulfide QD-containing film, recorded at 800 nm excitation.

In Figure 5, the Raman spectrum of the lead sulfide QD-containing film is shown, in the 100–1500 cm^−1^ domain. The Raman maxima could be attributed to the lead sulfide semiconductor nanoparticles and to the Si–O and P–O vibration modes.

The three Raman bands from the 235–460 cm^−1^ region are attributed to the phonon modes of PbS [38]. Consequently, the peak at 230 cm^−1^ is assigned to the longitudinal optical mode (LO) of PbS [38], the peak at 283 cm^−1^ is assigned to lead sulfide molecules [39,40], and the band at 352 cm^−1^ is assigned to Pb oxide species [38]. The band from about 454 cm^−1^ is allotted to the first overtone of the LO of PbS (2LO) [38], together with δ(Si–O–Si) [41]. The high intensity peak from 553 cm^−1^ is assigned to γ(Si–O–Si) [42]. Low-intensity maxima are found at 781 cm^−1^, attributed to σ_sym_(Si–O–Si) [43], σ(Si–O–R), and TEOS [44] and at 944 cm^−1^, allotted to σ(Si–O−) [42]. A high-intensity peak is found at 1092 cm^−1^, which is assigned to σ_asym_(Si–O–Si) [36], σ_asym_(SiO_4_)^4−^ [43], and σ(Si–O–P) [44]. (Note: σ, stretching vibration mode; δ, bending vibration mode; and γ, rocking vibration mode.)

### 3.4. Field Emission Scanning Electron Microscopy and Energy Dispersive X-ray Analysis (FESEM-EDX)

**Figure 6:**
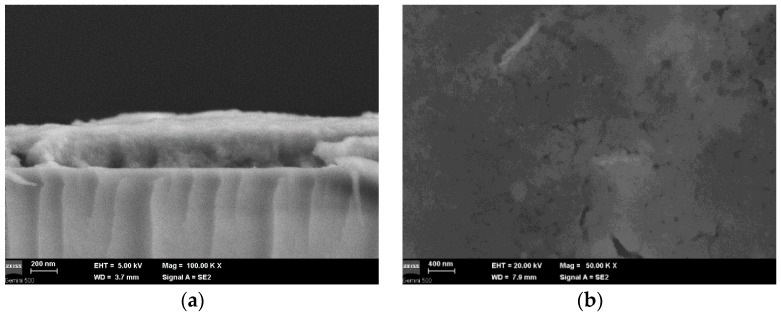
(**a**) FESEM image in the cross section of the lead sulfide QD-containing film coated on a glass substrate; (**b**) FESEM image of the lead sulfide QD-containing film surface.

In the FESEM image presented in Figure 6a, the cross section of the lead sulfide QD-containing film is shown, at a 100,000 magnification. The average thickness of the film was assessed to be 325 nm. The FESEM image of the film surface at 10,000 magnification is revealed in Figure 6b.

Film pores can be observed, which are specific to the sol-gel synthesis route. Some cracks are observed in the film owing to the drying stage, as well as to water and alcohol evaporation from the host matrix.

**Figure 7:**
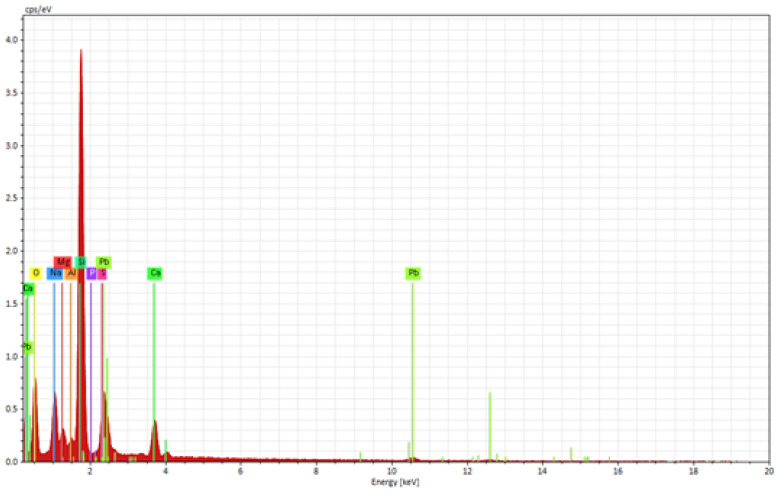
EDX elemental composition of the PbS QD-containing film coated on a glass substrate.

The elemental composition of the PbS QD-containing film coated on the glass substrate was evaluated using EDX analysis (Figure 7 and Table 1).

The EDX analysis revealed the presence of elements characteristic to both the deposited film and the glass substrate, like Si, Na, Mg, Ca, and O, as well as elements characteristic to the coated film, like Al, P, Pb, and S.

### 3.5. Transmission Electron Microscopy (TEM)

**Figure 8:**
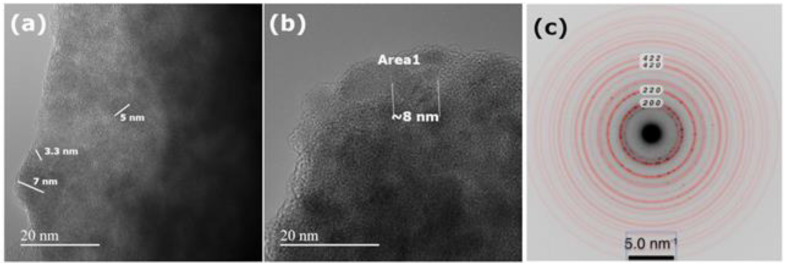
(**a**,**b**) HRTEM images of PbS QD-containing film and (**c**) SAED pattern with superposed simulated diffraction pattern. The lattice fringes of a PbS nanocrystal along the crystallographic zone axis can be observed in Area 1.

High-resolution imaging on the PbS QD-containing film (Figure 8a,b) showed densely packed quasi-spherical nanocrystals, with a size below 10 nm. The selected area electron diffraction (SAED) pattern obtained on a large area (Figure 8c) was indexed with the cubic phase of PbS (Crystallography Open Database ID 9013402), cell length a, b, c being 5.92 nm.

Thus, it is possible to conclude that PbS QDs, with a size below 10 nm, can be corroborated both by the TEM analysis and by the quantum confinement effect based on the band gap energy deduced using two routes, namely, exciton binding energy-exciton peak energy and graphical determination. These PbS QD size values are near the value calculated by XRD analysis.

### 3.6. Atomic Force Microscopy (AFM)

The AFM characteristics that were observed provide important information about the peculiarities of the layer and the surface roughness. The average squared roughness value (*R_q_*) signifies the standard deviation of the height value in the chosen region and *Max* is the maximum height value of the region.

The layer’s surface was scanned in non-contact mode, and the roughness was calculated for the greatest area of 40 µm × 40 µm.

**Figure 9:**
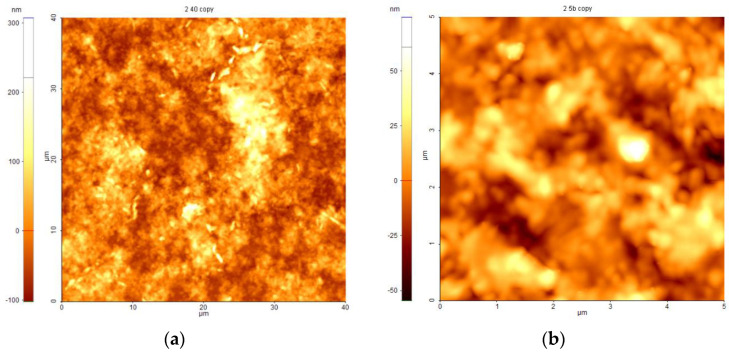
AFM images of the lead sulfide QD-containing layer’s surface, scanned on various areas: (**a**) 40 μm × 40 μm; (**b**) 5 μm × 5 μm.

The layer completely covers the substrate; no cracks or large agglomerations are present in a large area (40 µm × 40 µm). The high roughness value (*R_q_*∼49 nm) designates a nanostructured surface, as can be observed in Figure 9a. Nanostructures as small “grains” that can be assigned to the semiconductor cluster and create different structures having elongated shapes with sizes between 1.8 µm and 4.5 µm as well as round island shapes with sizes of approximately 1 µm. The maximum height of the biggest nano aggregate is about 310 nm.

The values of *R_q_* (49.1 nm) and *Max* (307.6 nm) certify the layer’s smoothness at a microscopic scale. In order to observe some surface details, a 5 μm × 5 μm area was scanned (Figure 9b). The nanostructured surface reveals small grains with dimensions of approximately 200–400 nm.

## 4. Conclusions

A PbS QD-containing film was synthesized by using the sol-gel route combined with the spin coating technique. The lead sulfide nanocrystalline phase and the amorphous phase as a host inorganic network were analyzed. Lead sulfide QDs with dimensions ranging from 2.78 nm to 14.7 nm were observed. The band gap energy value calculated from the lead sulfide exciton peak binding energy was explored in relation to the band gap energy value that was graphically calculated from the absorption spectrum, using the Mott and Davis/Tauc law. The distinct values for the band gap energy calculated by applying the two methods are caused by the complex structure of the lead sulfide QD-containing film, taking into consideration that the Mott and Davis/Tauc law refers strictly to either amorphous or crystalline materials. The reliability of the graphical method for calculating the *E_g_* value was corroborated by the absorption spectrum fitting method (ASF), which showed close band gap wavelength values. The maximum emission of the PbS QD-containing film was close to that of the PbS QD precursor solution, in the near infrared range. Therefore, the photoluminescence characteristics were retained after the coating and drying stages of the PbS QD-containing film. The structural investigation highlighted the vibration modes specific to the Si–O–Si and Si–O–P bonds as well as to lead sulfide QDs. The morphological analyses of the lead sulfide QD-containing film revealed a somewhat porous network validated both by SEM and AFM investigations. The reduced film roughness confirmed the layer’s flatness at a microscopic scale with nanoclusters of tens of nanometers. The SEM investigation attested to the occurrence of characteristic elements for the coated film, like Al, P, Pb, and S, as well as the elements O and Si, both belonging to the amorphous network and the lead sulfide QDs-containing film. The size of the PbS QDs ranged below 10 nm, inferred both by the quantum confinement effect based on the energy band gap value and the TEM analysis, close to the size value inferred by XRD analysis.

Our research demonstrates the possibility of using a green technology to fabricate homogeneous PbS QD-containing film with a nanostructured porous structure, where precise control of the size and spatial distribution of the PbS QDs plays a key role for applications. The known temperature dependence of PbS QD luminescence was demonstrated to be preserved in the film, thus making it a potential candidate for non-contact temperature-sensing devices.

## Figures and Tables

**Table 1:** The elemental composition of the PbS QD-containing film coated on a glass substrate.

Element	Mass (%)	Atomic (%)	Abs. Error (%)
O	44.4	59.13	12.58
Si	30.24	22.94	3.20
S	0.54	0.36	0.12
Al	0.97	0.76	0.19
Pb	2.99	0.31	0.29
Na	10.81	10.02	1.76
Mg	3.27	2.87	0.51
Ca	6.79	3.61	0.56

## Data Availability

Data are contained within the article.
